# Bidirectional Relationship between Positive Parenting Behavior and Children’s Self-Regulation: A Three-Wave Longitudinal Study

**DOI:** 10.3390/bs14010038

**Published:** 2024-01-05

**Authors:** Su Wang, Xiaosong Gai

**Affiliations:** 1 School of Psychology, Northeast Normal University, 5268 Renmin Street, Changchun 130024, China; wangs427@nenu.edu.cn; 2 Research Center of Mental Health Education in Northeast Normal University, Key Research Institute of Humanities and Social Science in Universities in Jilin Province, Changchun 130024, China

**Keywords:** positive parenting behavior, self-regulation, inhibitory control, autonomy support, cross-lagged design

## Abstract

In this study, we used a cross-lagged design to explore the relationship between children’s self-regulation and positive parenting behaviors. Children aged 3 years (N = 84) were tested individually three times a year for their hot and cool self-regulation, while their parents’ positive parenting behaviors (warmth, structure, and autonomy support) were collected through questionnaires. In the structural equation panel model, bidirectional relations between children’s inhibitory control and parental positive parenting were found. Children’s inhibitory control and positive parenting predicted changes in each other for the first six months. Such a reciprocal relationship also existed between parental autonomy support and children’s inhibitory control. There was a cross-lagged effect between parental warmth and children’ inhibitory control rather than a simultaneous relation. Children’s inhibitory control positively predicted parental structural behaviors rather than vice versa. Children’s delayed waiting and positive parenting (autonomy support) were only positively correlated, rather than having a lagging effect. All the relationships faded over time.

## 1. Introduction

Self-regulation is the process by which individuals actively regulate their emotions, motivations, behaviors, and cognitive arousal to achieve optimal levels of adaptation [1]. Self-regulation is an important aspect in the development of children’s social abilities. Early childhood is a period of rapid development of self-regulation, with an important turning point at the age of 4 [2,3].

Parenting plays an important role in shaping the development of self-regulation during early childhood. However, theories and research prove that parenting does not affect children in isolation, and children’s behaviors might shape or evoke parenting behaviors. Based on the ideas of L.S. Vygotsky and A. R. Luria, psychological processes are systems in constant development. Activities and social interaction lead to higher levels of self-regulation. This self-regulation does not manifest from the beginning, but is an ideal and dialectical possibility with potential for cultural development under certain conditions [4]. Family systems theory asserts that family processes are interdependent across family members—mother, father, and child—while transactional models propose that parenting and child characteristics are mutually influential [5]. Moreover, growing empirical evidence has shown that parents and children influence each other’s behaviors bidirectionally over time. In a meta-analysis study, the results showed that parenting is associated with children’s self-control both concurrently and longitudinally. Longitudinal studies have also revealed that children’s self-control influences subsequent parenting [6]. In a three-wave longitudinal study, bidirectional relationships between intrusive parenting and effortful control were found: children’s effortful control predicted lower intrusive parenting a year later, controlling for prior levels of parenting, and vice versa [7]. Additionally, a bidirectional relationship exists between harsh parenting styles and children’s self-regulation. Harsher parenting predicts lower self-regulation in children. Conversely, higher self-regulation predicts less harsh parenting [8]. However, some studies have found that parents’ higher structure and rule setting predict lower self-regulation difficulties in adolescents after 8 months, but children’s initial self-regulation cannot predict later parenting. There is no cross-lagged relationship between parental responsiveness and children’s behavior [9]. In a study aimed at children’s temperament and maternal parenting behavior, the relationships among self-regulation, parental acceptance, and parenting inconsistencies appeared to be simultaneous rather than predictive [10]. Eisenberg and colleagues (2005) conducted a longitudinal study of 186 adolescents three times, each two years apart. The results proved that warm expressions from parents positively predicted children’s effortful control after two years, but warm expressions of 11-year-olds could not predict children’s effortful control at 13 years old. An effect of children’s effortful control on parents’ warm expressions did not appear [11]. A bidirectional relationship exists between children’s effortful control and authoritarian parenting, whereas no significant correlation exists between children’s effortful control and authoritative parenting [12]. Moilanen and colleagues (2015) also found that children’s self-regulation had a bidirectional effect on authoritarian parenting and a predictive effect on permissive-indulgent parenting, but no cross-lagged effect on authoritative parenting [13]. Many studies have found that authoritarian or controlled parenting can significantly stimulate children’s affective overarousal and result in more self-regulation difficulties [11]. Research on the relationship between positive parenting and children’s self-regulation can improve family education guidance and promote positive development in children. These studies on parents’ positive parenting behavior either select a single parenting behavior [11] or integrate responsiveness, autonomy support, and positive behavior control into a global construct, authoritative parenting [12,13], which obscures the relationship between different parenting behaviors and aspects of children’s self-regulation [11]. Two reasons below may explain this conflict.

This bidirectional relationship may differ because of positive parenting indicators. According to self-determination theory and the results of studies on parenting, positive parenting behavior refers to warmth, positive behavior control, and autonomy support [14]. Parental warmth, positive behavior control, and autonomy support can promote children’s self-regulation, with autonomy support having the strongest effect [15]. Positive behavioral control promotes self-regulation, whereas responsive parenting is weak [16]. Mothers’ encouragement of autonomy and toddlers’ cooperative obedience promote each other, whereas mothers’ sensitivity and mind-mindedness do not [17]. Blair and colleges (2014) used various methods to determine whether there was a bidirectional relationship between children’s executive function and responsive parenting. Parental responsiveness predicts changes in children’s executive function. Executive functioning also predicts changes in parental responsiveness. However, executive function can predict changes in sensitive parenting, while parental sensitiveness cannot predict changes in executive function [18]. The unique bidirectional relationship of each parenting dimension may not be apparent unless a comprehensive set of parenting dimensions is included in the same study. Such a bidirectional relationship may exist among parental autonomy support, responsiveness, positive parenting behavior, and children’s self-regulation during early childhood.

The type of self-regulation task also affected this relationship. Parenting behavior is significantly associated with compliance, but not with inhibitory control or emotional regulation [16]. Vansteenkiste and colleagues (2014) found that the relationship between parental autonomy support and children’s introjected regulation is bidirectional, but that there is no such effect between autonomy support and other motivations [19]. Parental autonomy support predicted higher introjected regulation, but not vice versa. Parental autonomy support did not predict external regulation; however, higher external regulation predicted lower autonomy support [20]. There was a bidirectional relationship between mothers’ negative expressions and their children’s effortful control in a study by Klein and colleagues (2018). Negative expressions of mothers of 36-month-old children can predict lower effortful control in 54-month-old children, whereas children’s effortful control can also predict less negative expression in the mother. In addition to responsiveness, mothers’ warmth, limited setting, and scaffolding predicted children’s stronger effortful control, but not vice versa. However, there was no relationship between the mothers’ parenting behaviors and their children’s delayed waiting ability [21]. However, some studies have found that parental responsiveness can predict children’s executive function (delayed waiting and conflict control) after six months, but only delayed waiting can predict parental responsiveness after six months, while conflict inhibition cannot predict parenting behavior [22]. Different components of children’s self-regulation have different relationships with positive parental behavior [21]. Self-regulation has been proven to be a two-factor structure with hot and cool aspects [2,23]. Behavioral genetics analyses have shown that individual differences in hot and cool EC can mostly be explained by environmental factors [24]. Parenting may be contributing differently to hot and cool EF development [25]. It is necessary to demonstrate the relationship between these aspects and positive parenting behaviors.

The current study is based on classical theories like family systems theory and transactional models emphasizing bidirectional relationships between parents and children. Structural equation modeling can be used to test the plausibility of causal associations, especially when the data are longitudinal and the key variables are tested at three or more time points [11]. Therefore, using a three-wave cross-lagged longitudinal design with an interval of six months, this study investigated 3-year-olds and their parents to explore the relationship between different positive parenting behaviors (warmth, autonomy support, and structure) and the components of young children’s self-regulation (inhibitory control and delayed waiting) to guide education on improving young children’s self-regulation. Based on the results of a previous study, the following hypotheses were proposed: (1) Positive parenting behaviors predict children’s self-regulation, and vice versa. (2) Such a reciprocal relationship exists between parental autonomy support and inhibitory control.

## 2. Materials and Methods

### 2.1. Sample

Participants were preschool-aged children and their families, attending four large kindergartens in a city in China, from a longitudinal study (at T1, N = 320, 159 males, 161 females, mean age 41.91 months, age range 32–49 months). Due to the reduction in effective questionnaires for parents and the low attendance rate of children, the parent–child dyads numbered 151 at T2 after six months. After another six months, 84 children (44 males, 40 females, mean age 41.88 months, age range 34–48 months) were measured in individual tests, and their parents reported their positive parenting behaviors all three times. Subjects in this study, compared to overall subjects, did not differ significantly in every aspect regarding age (t (310) = −0.101, *p* > 0.05), inhibitory control (t (83) = 0.990, *p* > 0.05), delayed waiting (t (83) = 0.383, *p* > 0.05), or parenting behaviors (warmth t (83) = 1.145, autonomy support t (83) = 0.634, and structure t (316) = 0.864, *ps* > 0.05). None of the children had sensory, behavioral, or psychological developmental difficulties, and all had normal or corrected vision. Based on the region in which the kindergartens were located, the socioeconomic statuses of the families in which the children lived were moderate.

### 2.2. Measures

#### 2.2.1. Self-Regulation

Day–night task [26]: Gerstadt, Hong, and Diamond’s (1994) “Day–Night” (D–N) Stroop task was adopted. The materials included 16 cards measuring 10 cm × 15 cm, including light-blue cards depicting the sun and white clouds and black cards depicting the moon and stars. The task required children to say “night” when they saw pictures of the sun and “day” when they saw pictures of the moon and stars. After the rules were explained, the children conducted four exercises that were corrected when they gave incorrect answers. After ensuring that the participants answered correctly two or more times, they were administered 16 formal tests. The cards were presented eight times in random order, and without correction for errors. Half of the cards corresponded to each type. The scores were 0, 1, and 2, with 0 = incorrect (wrong or no answer); 1 = incorrect at the beginning, correct at the end, or correct after hesitation; and 2 = correct immediately. Complex tasks were included in the second test. There were sixteen trials, of which eight were non-moving, four were days, and four were nights. The scoring was the same as that in the simple task.

Head-to-toes task [27]: The head-to-toes task by McClelland et al. (2007), which consisted of an exercise phase and a formal phase, was adopted. The participants were asked to execute the “reverse action.” When they heard “touch your head”, they touched their feet; when they heard “touch your toes”, they touched their heads. After two correct exercises, a formal test was conducted without further correction. Head-touch and toe-touch were presented 16 times at random, and the score of each response was recorded. The scores were 0, 1, and 2, with 0 = incorrect (incorrect response or no response), 1 = tendency to start incorrectly and end up responding correctly or to hesitate for a while before responding correctly, and 2 = correct (responding without hesitation). A complex task was added to the third test (see the day–night task). There were 16 trials, of which 8 were non-moving, 4 were head-touching, and 4 were toe-touching. The scoring was the same as that in the simple task.

The combined average score of the day–night task and head-to-toes task was the IC score of inhibitory control, ranging from 0–2.

Gift delay task [22,28]: The gift warp/delay task of Kochanska et al. (1996) was adopted. The materials included several small gifts and plastic wrapping paper. Children were told that they would be receiving a present, but that they could not peek while the present was being wrapped. Subsequently, the children were instructed to turn their backs to the examiner as the examiner noisily wrapped the present around them. They were asked to wait for two minutes (120 s) to see whether the children would peek. If they turned around and peeked, the latency time was recorded immediately and the task was stopped. If they peeked once, the latency time was recorded and a reminder was provided (if they peeked again, they did not receive the gift). If they peeked twice, the game was stopped and the time was recorded. Children who completed the task chose small gifts. The scores included (a.) whether to peek: 0 = turn around or peek twice, 1 = peek once, and 2 = no peek; and (b.) latency time: the time from when the subject began wrapping the gift to when the child first peeked. The scores were combined into a score for delayed waiting, ranging from 0 to 2. The scores for strategy and latency to peek were averaged to create the delayed waiting total score.

#### 2.2.2. Positive Parenting Behavior

The positive parenting behavior questionnaire was adapted from the scale of parenting [29]. The questionnaire included three dimensions—warmth, structure, and autonomy support—with 21 questions in total. The questionnaire was scored on a five-point scale, with 1 = not met at all and 5 = fully met. The higher the score, the more positive the parenting behavior. Warmth consisted of 8 questions, with a consistency coefficient of 0.781–0.820; autonomy support, 10 questions and a consistency coefficient of 0.826–0.861; and structural behavior, 3 questions and a consistency coefficient of 0.570–0.632. The questionnaire was found to be reliable.

### 2.3. Procedure

All study procedures were approved by the Ethics Committee of the School of Psychology, Northeast Normal University (protocol code 201603). For the first wave, informed consent was obtained from parents for themselves and their children through teachers in the kindergartens. Children were assessed for inhibitory control and delayed waiting for the first time during the fall semester. After a six-month interval, the children were followed up for the second time the following spring. After another six-month interval, a third follow-up test was performed following the fall. Children were measured in quiet spare rooms at their kindergartens. The participants were undergraduate and graduate students majoring in preschool education and psychology. No child spent more than 30 min on the test.

The parenting questionnaire was issued to parents by the teacher during the individual test period and was collected before the end of the test at one-week intervals.

### 2.4. Analysis

First, we examined the correlations among variables using SPSS 22.0. Subsequently, we used structural equation modeling to evaluate the statistical model using the Amos 22.0. to evaluate the fit of the structural model to the data, the standard chi-square index, the root mean square error of approximation (RMSEA), and the comparative fit index (CFI). RMSEA is an absolute index of fit, with values less than 0.05 indicating a close fit to the data. For the CFI, fit index values should be greater than 0.90 to consider the fit of a model to the data to be acceptable [11].

## 3. Results

### 3.1. Preliminary Results

Investigating the data distribution of indicators of children’s self-regulation and positive parenting behavior, the results showed that most indicators were normally distributed, with cutoff values of 2 for skewness and 7 for kurtosis [11,30]. Only children’s inhibitory control at T3 was skewed (skewness = −3.137), which may indicate a ceiling effect.

To examine the relationship between children’s self-regulation and positive parenting behavior, we first examined the correlations between the variables (Table 1).

The results showed that children’s inhibitory control was significantly correlated to positive parenting at all times (except for the correlation between warmth and inhibitory control at T1, which was not significant; r = 0.089). Children’s delayed waiting was not significantly correlated with most positive parenting indicators (except that delayed waiting at T1 was correlated with autonomy support and positive parenting, *rs* = 0.216, 0.236, *ps* < 0.05; delayed waiting at T2 was significantly correlated to warmth and autonomy support, *rs* = 0.224, 0.277, *ps* < 0.05). This suggests that the relationships between the different components of children’s self-regulation and parents’ positive parenting behaviors were slightly different. Positive parenting behaviors were significantly related to children’s inhibitory control, but not to delayed waiting.

### 3.2. Bidirectional Effects of Self-Regulation and Positive Parenting

Based on this analysis, children’s inhibitory control and positive parenting behaviors were found to be significantly related, and a cross-lagged model was used to examine whether there was a bidirectional relationship between self-regulation and positive parenting.

#### 3.2.1. Bidirectional Relationship of Inhibitory Control and Positive Parenting

Two comparative models were used to explore the relationship between inhibitory control and positive parenting.

**Figure 1:**
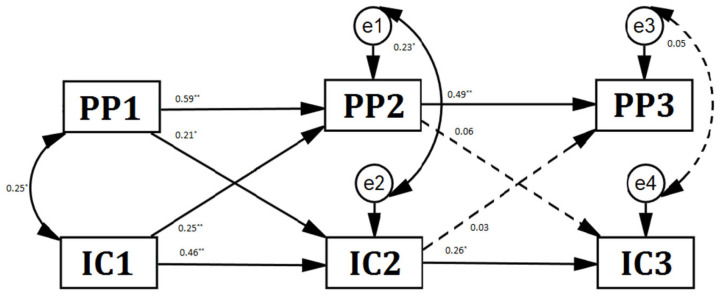
Results of structural equation model. Note: IC = inhibitory control, PP = positive parenting, * *p* < 0.05, ** *p* < 0.01. Results are standardized coefficients. Dotted lines are nonsignificant paths.

In Model 1, we constrained all paths between parenting and inhibitory control from T1 to T2 as invariant, with comparable paths between the constructs from T2 to T3. Thus, the correlation coefficient between parenting and child inhibitory control at T2 was controlled to be equal to that between parenting and inhibitory control at T3. Model 1 had quite the acceptable fit to the data, although the statistical index of fit showed a significant model misfit: *χ*^2^(9) = 20.622, *χ*^2^*/df* = 2.291, CFI = 0.902, RMSEA = 0.125. In Model 2, we fit a free estimation model. Model 2 had a quite acceptable fit to the data: *χ*^2^(4) = 6.292, *χ*^2^*/df* = 1.573, CFI = 0.981, *RMSEA* = 0.083. Furthermore, the difference in fit between Models 1 and 2 was significant: Δ*χ*^2^(5) = 14.33 > 11.07, *p* < 0.05, indicating that the path coefficients from T1 to T2 differed significantly from comparable path coefficients from T2 to T3. We chose to retain Model 2 as our final model, as illustrated in Figure 1.

As depicted in Figure 1, positive parenting was quite stable over time, whereas child inhibitory control was moderately stable. Children’s inhibitory control and positive parenting were simultaneously related (*rs* = 0.25, 0.23, *ps* < 0.05) at T1 and T2. Children’s inhibitory control and positive parenting were bidirectionally predictive from T1 to T2. Positive parenting at T1 predicted children’s inhibitory control at T2 (*β* = 0.21, *p* < 0.05), while children’s inhibitory control at T1 predicted positive parenting at T2 (*β* = 0.25, *p* < 0.05). However, such a bidirectional relationship disappeared from T2 to T3 (*β*s = 0.06, 0.03, *ps* > 0.05). All the relationships between inhibitory control and positive parenting faded over time.

Specifically, the bidirectional relationship between children’s inhibitory control and positive parenting mainly manifested in autonomy support and warmth. The model of autonomy support and inhibitory control fit the data well (details in Table 2 and Table 3). Children’s inhibitory control was correlated with autonomy support at both T1 and T2 (*rs* = 0.25, 0.30, *ps* < 0.05). Moreover, children’s inhibitory control and positive parenting were bidirectionally related at T1 and T2. That is, inhibitory control at T1 predicted autonomy support at T2 (*β* = 0.24, *p* < 0.05) and vice versa (*β* = 0.19, *p* < 0.05). This effect disappeared between T2 and T3 (*β*s = 0.01–0.02, *ps* > 0.05). The fit of the models of inhibitory control and warmth were also good (see Table 2 and Table 3). Children’s inhibitory control and parental warmth were not related simultaneously (*rs* = 0.089, 0.143, 0.060, *ps* > 0.05), but children’s inhibitory control and warmth predicted each other from T1 to T2. Parental warmth at T1 predicted inhibitory control at T2 (*β* = 0.21, *p* < 0.05), while inhibitory control at T1 also predicted warmth critically at T2, in reverse (*β* = 0.16, *p* = 0.054). Such cross-lagged effects did not appear between T2 and T3.

Furthermore, the models of inhibitory control and parental structure fit well (see Table 2 and Table 3). However, no bidirectional effects were observed. Child effects were also observed in the model. Children’s inhibitory control was significantly correlated with parental structure only at T1 (*r* = 0.25, *p* < 0.05). Children’s inhibitory control at T1 predicted structure at T2 (*β* = 0.30, *p* < 0.01), but not vice versa (*β* = 0.12, *p* > 0.05). As was the case for the other models, the relationship disappeared later (*β*s = 0.07, 0.03, *ps* > 0.05).

In short, the relationship between children’s inhibitory control and positive parenting was mutually causal and disappeared during mid-childhood. The higher the inhibitory control that children have, the more positive parenting the parents will exhibit. In turn, children demonstrated better inhibitory control performance.

#### 3.2.2. Bidirectional Relationship between Delayed Waiting and Positive Parenting

**Figure 2:**
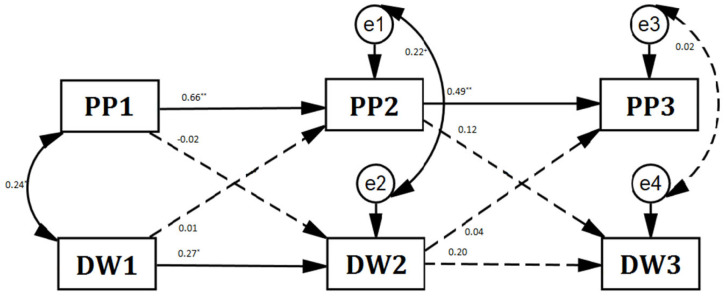
Cross−lagged model of positive parenting and delayed waiting. Note: DW = delayed waiting; PP = positive parenting; ^+^ *p* < 0.10, * *p* < 0.05, ** *p* < 0.01.

To explore the relationship between delayed waiting and positive parenting, a cross-lagged analysis was conducted using a free-estimation model, as depicted in Figure 2.

The model fit well: *χ*^2^ (4) = 1.601, *χ*^2^*/df* = 0.400, *CFI* = 1.000, *RMSEA* = 0.000. The results showed no cross-lagged effects between delayed waiting and positive parenting. Children’s delayed waiting times were simultaneously related to positive parenting at T1 and T2 (T1: r = 0.236, *p* < 0.05; T2: r = 0.217, *p* = 0.053). The relationship between delayed waiting and positive parenting behavior mainly manifested in autonomy support. Autonomy support at T1 and T2 was significantly associated with delayed waiting (T1: r = 0.216, *p* = 0.053; T2: r = 0.316, *p* < 0.01). Parental structure and warmth behavior had no synchronous correlation and no hysteresis effect with simultaneous delayed waiting (structure, *rs* = 0.207, 0.023, 0.060, *ps* > 0.05; warmth, *rs* = 0.14, 0.220 ^*^, 0.019, *ps* ≥ 0.05). These results indicate that delayed waiting has little longitudinal influence on parenting, and vice versa. No cross-lagged causal relationship exists between delayed waiting and parenting.

## 4. Discussion

In this study, we used a three-wave longitudinal design to explore the relationship between children’s self-regulation and positive parenting. First, the results elucidated a bidirectional relationship between children’s inhibitory control and positive parenting. According to Vigotsky’s theory, the process of psychological development passes from the stage of regulation of a child’s activity by an adult’s external rules to the stage of regulation of a child’s own rules. The most important means is language. As an adult uses language for the regulation of a child, the child uses their own external language for self-regulation. Later on, the child might be able to regulate self-activity using internal language [31]. Positive parenting makes it possible to transfer warm and structural language and rules in an autonomy-supportive way to improve children’s self-regulation effectively. These findings suggest that in early childhood, or even earlier, positive parenting behaviors can promote children’s ability to suppress dominant responses and activate a subdominant response, plan, and detect errors, which enable parents to use more positive parenting behaviors. However, as the children age, this relationship weakens or even disappears. This may be due to the following reasons. First, children’s self-regulation is affected by genetic background, prenatal care, nutrition, and brain development, and develops rapidly in early childhood, resulting in weaker stability [22]. Second, possibly, the effects of positive parenting are especially strong in the earlier years, when children are more vulnerable and parents are highly salient socializers compared to other socialization influences such as teachers and peers [11]. After children enter kindergarten and they interact more with teachers and peers, their relationship with their parents changes, and their communication time decreases. Thus, the interaction effect between children and parents is weakened. Third, owing to the bias of the inhibitory control task in T3, the data appeared to have a ceiling effect in the later period; therefore, the variation became smaller, resulting in a weak correlation. Fourth, it is possible that different forms of measurement, such as questionnaires on parenting and individual tests of self-regulation, lead to different trends that may contribute to the weakening of the relationship between parenting and SR. This also suggests that researchers should consider studying the role of positive parenting or improving positive parenting interventions in early childhood.

Second, distinguishing between different positive parenting behaviors, the relationship between young children’s self-regulation and autonomy support is representative. Parental autonomy support was found to be mutually causal with children’s inhibitory control and was simultaneously related to delayed waiting. One possible reason for this is that, according to SDT, parental autonomy support can provide effective scaffolding support for children to transition from external control to self-control and meet their psychological needs in order to improve their cognitive abilities [32]. Concurrently, parental autonomy support behavior is highly malleable [33] and influenced by young children’s temperaments, behavioral performances [34,35,36], and knowledge about parenting and child development [37,38]. These characteristics strengthen and stabilize the correlation between self-regulation and parental autonomy. Although Chinese parents show less autonomy-supportive parenting, the effect of autonomy support on child development is still significant, which confirms the cross-cultural universality of self-determination theory. Moreover, the reciprocal effect implies that, if a child has poor self-regulation performance, parents will provide less autonomy support, leading to more self-regulation difficulties. This vicious cycle prompts parents and teachers to pay attention to children with poor self-regulation at an early age and helps them improve their abilities to promote their development. It also prompts family education training to focus on improving parental autonomy support to optimize family education and promote children’s development.

Third, we found no significant relationship between children’s self-regulation and parental warmth behaviors. This is consistent with the results of a meta-analysis [16]. However, there was a cross-lagged relationship between parental warmth behavior and inhibitory control. Although parental warmth behavior was not related to children’s inhibitory control, at the same time, it predicted children’s inhibitory six months later. Children’s inhibitory control also predicted parental warmth behavior six months later. On the one hand, this may be because parents provide a warm and emotional family atmosphere that requires a long time to take effect. On the other hand, parental warmth may contribute to the formation of parent–child attachment, and secure attachment relationships play an important role in the development of children’s self-regulation [39]. So, we still need to keep warmth, sensitiveness, responsiveness, positive expressing, and positive reinforcement as a part of parenting projects.

Fourth, we found that children’s inhibitory control at age 3 could predict parental structure behaviors six months later, but there was no reverse effect. This finding suggests that the child has an effect on parenting. Compared to children who have internalized self-regulation, young children need more external rules and more language regulation to help them achieve self-regulation. According to Kopp’s model, children aged 12–18 months become aware of social demands and are able to comply with parental requests; children aged 2 have the ability to inhibit behavior and to regulate behavior even when parents are absent; and children aged 3 begin to be capable of self-regulation [16]. It is possible that the self-regulation of children aged 3–4 develops rapidly, and parents adjust their rules and behavioral controls on their children according to their children’s compliance. On the other hand, parent-structured rule-setting behaviors may not shape young children’s self-regulation as much as controlling parenting (harsh parenting [8]; authoritarian parenting [12,13]; or intrusive parenting [7]). Negative controlling parenting can significantly stimulate children’s affective overarousal and result in more self-regulation difficulties, whereas positive controlling behaviors cannot. This suggests that parents should adopt complicated behavioral control strategies that awaken children’s positive emotions, elaborate skill guidance, and provide optimal action, rather than simply providing limit-setting rules. Moreover, when facing children with poor self-regulation, parents must avoid chaotic and emotionally controlling behavior and maintain stable and calm behavior to reduce the negative impact on children.

Fifth, the results showed that delayed waiting was only correlated with positive parenting (autonomy support) simultaneously, and not in a cross-lagged manner. For instance, delayed waiting for young children did not predict positive parenting behaviors. This is in contrast to the results reported by Merz’s study. The reason may be that parenting in Merz’s study was coded according to parent–child free play videos, while it was measured with a questionnaire in this study, which indicated more global positive parenting. This is similar to the results of Klein and colleges (2018), who showed that positive parenting behavior did not predict children’s delayed waiting. As children’s performance in delayed waiting tasks reflects their impulse control or ability to withhold automatic responses in the context of a reward, it is easily affected by situational factors, such as emotional state, affection for rewards, and test environment. Therefore, responsive or reactive aspects of parenting may be related to children’s delayed waiting abilities. Some individual factors, such as gender [23,40], general IQ and emotion knowledge [40], and temperament [22], may significantly affect children’s delayed waiting abilities.

In this study, some limitations exist, and the results should interpreted with caution. First, the number of subjects in this study was small. It is necessary to increase the number of subjects to validate the research results. Second, parenting was measured using a questionnaire, and the structural parenting dimension had only three questions, with a consistency coefficient of around 0.6. Therefore, future studies should adopt both questionnaires and observations to obtain more objective and comprehensive indicators of parenting behavior. Finally, the study tracked the relationship between self-regulation and parenting in children aged 3–4 years and concluded that the relationship may be more important at earlier ages. Therefore, future studies should be extended to younger infants.

## 5. Conclusions

In this study, we examined the relationship between children’s self-regulation and parents’ positive parenting behaviors using a three-wave cross-lagged design method. The results demonstrated that there was a bidirectional relationship between children’s inhibitory control, autonomy support, and parental warmth. Children’s inhibitory control can affect parental structured behavior, and there is no reverse effect. There was a simultaneous correlation between delayed waiting and positive parenting behaviors. All relationships diminished with increasing age. These results suggest that we should pay attention to training in positive parenting behavior for parents of young children, particularly concerning autonomy support.

## Figures and Tables

**Table 1:** Zero-order correlations between children’s self-regulation and parental positive parenting behavior.

	M/SD	1	2	3	4	5	6	7	8	9	10	11	12	13	14	15	16	17	18
1. IC1	0.938/0.444	1																	
2. IC2	1.435/0.388	0.514 **	1																
3. IC3	1.773/0.234	0.310 **	0.290 **	1															
4. DW1	1.058/0.830	0.484 **	0.154	0.297 **	1														
5. DW2	1.590/0.622	0.226 *	0.249 *	0.040	0.270 *	1													
6. DW3	1.648/0.629	0.085	0.062	0.252 *	0.155	0.224 *	1												
7. W1	3.975/0.548	0.089	0.256 *	0.039	0.140	0.103	0.148	1											
8. W2	3.991/0.550	0.215 *	0.331 **	0.191	0.071	0.224 *	0.134	0.640 **	1										
9. W3	3.955/0.574	0.219 *	0.204	0.153	0.071	0.153	0.089	0.461 **	0.477 **	1									
10. AS1	4.132/0.488	0.247 *	0.309 **	0.142	0.216 *	0.048	0.130	0.769 **	0.623 **	0.320 **	1								
11. AS2	4.166/0.485	0.368 **	0.483 **	0.124	0.118	0.277 *	0.125	0.416 **	0.699 **	0.345 **	0.569 **	1							
12. AS3	4.190/0.455	0.325 **	0.247 *	0.133	0.132	0.093	0.035	0.322 **	0.505 **	0.683 **	0.436 **	0.500 **	1						
13. S1	3.637/0.819	0.255 *	0.244 *	0.145	0.207	0.028	0.059	0.249 *	0.305 **	0.141	0.477 **	0.373 **	0.151	1					
14. S2	3.857/0.681	0.412 **	0.374 **	0.137	0.153	0.046	0.133	0.227 *	0.414 **	0.265 *	0.457 **	0.639 **	0.377 **	0.521 **	1				
15. S3	3.818/0.717	0.314 **	0.192	0.071	0.155	0.101	0.120	0.091	0.262 *	0.425 **	0.289 **	0.231 *	0.572 **	0.248 *	0.360 **	1			
16. PP1	3.915/0.497	0.254 *	0.329 **	0.140	0.236 *	0.069	0.129	0.756 **	0.606 **	0.351 **	0.871 **	0.544 **	0.344 **	0.797 **	0.519 **	0.264 *	1		
17. PP2	4.005/0.484	0.398 **	0.462 **	0.178	0.138	0.199	0.155	0.488 **	0.807 **	0.420 **	0.640 **	0.899 **	0.535 **	0.485 **	0.840 **	0.345 **	0.655 **	1	
18. PP3	3.988/0.487	0.342 **	0.252 *	0.136	0.145	0.139	0.105	0.327 **	0.474 **	0.815 **	0.404 **	0.405 **	0.861 **	0.224 *	0.398 **	0.836 **	0.375 **	0.502 **	1

Note: IC = inhibitory control, DW = delayed waiting, W = warmth, AS = autonomy support, S = structure, PP = positive parenting, * *p* < 0.05, ** *p* < 0.01.

**Table 2:** Model fitting indices of positive parenting and children’s self-regulation.

	χ^2^	df	χ^2^/df	p	CFI	RMSEA	90% CI RMSEA
Model (PP-IC)	6.292	4	1.573	0.178	0.981	0.083	0.000–0.209
Model (W-IC)	10.651	4	2.663	0.031	0.937	0.142	0.039–0.248
Model (S-IC)	6.987	4	1.747	0.137	0.964	0.095	0.000–0.209
Model (AS-IC)	10.169	4	2.542	0.038	0.944	0.136	0.030–0.243
Model (PP-DW)	1.601	4	0.400	0.809	1.000	0.000	0.000–0.103
Model (W-DW)	6.042	4	1.511	0.196	0.972	0.078	0.000–0.197
Model (S-DW)	1.571	4	0.393	0.814	1.000	0.000	0.000–0.102
Model (AS-DW)	5.459	4	1.365	0.243	0.979	0.066	0.000–0.189

Note: PP = positive parenting, IC = inhibitory control, DW = delayed waiting, W = warmth, AS = autonomy support, S = structure.

**Table 3:** Coefficients of cross-lagged models.

	P (T1–T2)	C (T1–T2)	Parent T1–Child T2	Child T1–Parent T2	P (T2–T3)	C (T2–T3)	Parent T2–Child T3	Child T2–Parent T3
Model (W-IC)	0.63 (0.08) **, 0.63	0.43 (0.08) **, 0.50	0.15 (0.07) *, 0.21	0.20 (0.10) +, 0.16	0.48 (0.11) **, 0.46	0.15 (0.07) *, 0.26	0.05 (0.05), 0.11	0.08 (0.15), 0.05
Model (S-IC)	0.37 (0.08) **, 0.45	0.42 (0.08) **, 0.48	0.06 (0.05), 0.12	0.46 (0.14) **, 0.30	0.35 (0.12) **, 0.34	0.17 (0.07) *, 0.28	0.01 (0.04), 0.03	0.13 (0.20), 0.07
Model (AS-IC)	0.51 (0.09) **, 0.51	0.41 (0.08) **, 0.47	0.15 (0.08) *, 0.19	0.27 (0.10) **, 0.24	0.47 (0.10) **, 0.50	0.18 (0.07) *, 0.30	−0.01 (0.06), −0.02	0.01 (0.13), 0.01
Model (W-DW)	0.65 (0.09) **, 0.64	0.20 (0.08) *, 0.26	0.08 (0.12), 0.07	−0.01 (0.06), −0.02	0.49 (0.10) **, 0.47	0.21 (0.11), 0.20	0.10 (0.13), 0.09	0.05 (0.09), 0.05
Model (S-DW)	0.43 (0.08) **, 0.51	0.21 (0.08) *, 0.28	−0.02 (0.08), −0.03	0.04 (0.08), 0.05	0.38 (0.11) **, 0.36	0.22 (0.11) *, 0.22	0.11 (0.10), 0.12	0.10 (0.12), 0.09
Model (AS-DW)	0.57 (0.09) **, 0.57	0.20 (0.08) *, 0.27	−0.01 (0.14), −0.01	−0.00 (0.05), −0.01	0.48 (0.09) **, 0.51	0.21 (0.11), 0.21	0.09 (0.14), 0.07	−0.04 (0.07), −0.05

Note: IC = inhibitory control, DW = delayed waiting, W = warmth, AS = autonomy support, S = structure, ^+^ *p* < 0.10, * *p* < 0.05, ** *p* < 0.01. Results are non-standardized coefficients with standard errors in parentheses and standardized coefficients.

## Data Availability

The data that support the findings of this study are contained within the article and are available from the corresponding author upon reasonable request.
